# Eliminating biasing signals in lung cancer images for prognosis predictions with deep learning

**DOI:** 10.1038/s41746-019-0194-x

**Published:** 2019-12-10

**Authors:** W. A. C. van Amsterdam, J. J. C. Verhoeff, P. A. de Jong, T. Leiner, M. J. C. Eijkemans

**Affiliations:** 1 Department of Radiology, University Medical Center Utrecht, Utrecht University, Utrecht, The Netherlands; 2 Department of Radiation Oncology, University Medical Center Utrecht, Utrecht University, Utrecht, The Netherlands; 3 Biostatistics and Research Support, Julius Center for Health Sciences and Primary Care, University Medical Center Utrecht, Utrecht University, Utrecht, The Netherlands

**Keywords:** Prognosis, Computer science, Epidemiology, Computed tomography

## Abstract

Deep learning has shown remarkable results for image analysis and is expected to aid individual treatment decisions in health care. Treatment recommendations are predictions with an inherently causal interpretation. To use deep learning for these applications in the setting of observational data, deep learning methods must be made compatible with the required causal assumptions. We present a scenario with real-world medical images (CT-scans of lung cancer) and simulated outcome data. Through the data simulation scheme, the images contain two distinct factors of variation that are associated with survival, but represent a collider (tumor size) and a prognostic factor (tumor heterogeneity), respectively. When a deep network would use all the information available in the image to predict survival, it would condition on the collider and thereby introduce bias in the estimation of the treatment effect. We show that when this collider can be quantified, unbiased individual prognosis predictions are attainable with deep learning. This is achieved by (1) setting a dual task for the network to predict both the outcome and the collider and (2) enforcing a form of linear independence of the activation distributions of the last layer. Our method provides an example of combining deep learning and structural causal models to achieve unbiased individual prognosis predictions. Extensions of machine learning methods for applications to causal questions are required to attain the long-standing goal of personalized medicine supported by artificial intelligence.

## Introduction

Deep learning has many possible applications in health care, especially for tasks including unstructured data such as medical images. Convolutional neural networks (CNN) are deep learning models that have demonstrated remarkable performance on many tasks including images. These models are attractive for prediction tasks on medical images, as CNNs can be optimized end-to-end from image to outcome. This way the network can detect patterns in the images that are relevant to the prediction task, but may be unknown to medical professionals. A downside is that the induced representations of the network are ‘hidden’ and not readily interpretable. A much sought after holy grail of artificial intelligence is to attain personalized treatment decisions through individual prognosis prediction and individual treatment effect estimation. Treatment effect estimation is a causal question, so answering it requires techniques from causal inference.^1^ A pivotal result from causal inference is that when the direction of causal relationships between variables in a given situation is known, identifiability and estimands of causal queries can be deduced automatically using do-calculus. In the case of treatment effect estimation of lung cancer measured with overall survival, this means that we must know (a) which variables affect both treatment allocation and overall survival, (b) the causal direction of relationships between the variables. For instance, we know that the level of pre-treatment overall fitness is related to the likelihood of getting intensive treatment. In this case the direction of causation is clear due to the time ordering: pre-treatment fitness influences the treatment decision, and not vice versa. Whether this is a strong or weak relationship, or the specific functional form of the relationship (e.g., whether the relationship is monotonic) is not important for the consideration of general non-parametric causal effect identification. These causal relationships can be encoded succinctly in a Directed Acyclic Graph (DAG) with an arrow pointing from the cause to the effect, e.g., ${\mathrm{fitness}}\to {\mathrm{treatment}}$. When the DAG that encodes the relationship between all the relevant variables is known, do-calculus provides an answer to whether a specific causal question can be answered from the observed data.

The connection between images and a DAG is not always straightforward to see. Fundamentally, patient outcomes are driven by biological processes, and images may contain (more or less noisy) views of these processes. For example, a particularly aggressive lung tumor may grow very large, as can be seen on CT-scans, and this biological behavior leads to worse overall survival. These biological processes can be seen as underlying causes of factors of variation or patterns in the image in the language of structural causal models. Conversely, information derived from medical images is often used to make treatment decisions. Here, the image is a causal factor for treatment selection. When a deep neural network is used to predict a certain clinical outcome, it will make use of all factors of variation in an image that are statistically associated with that outcome. Thus, predicting an outcome with deep learning based on an image can be seen as conditioning on (noisy views of) the underlying causal factors of the patterns in these images. Medical images, especially images from large body parts such as a chest CT-scan in the case of lung cancer, may contain many different factors of variation that can have different ‘roles’ in the DAG. Notably when a specific factor of variation represents a collider in the DAG, conditioning on the image by using a deep learning model may introduce bias in the estimation of treatment effects.

A collider is a variable that is the effect of two or more variables. To explain collider bias, consider the following clinical scenario. The pulmonary oncology department in a general hospital serves the population of a small geographic region for all cases of lung cancer, and 90% of their patients come from this region. However, one of the oncologist has a special interest in the treatment of a rare form of lung cancer: carcinoid tumors, accounting for roughly 1% of lung cancer cases. Everyone in the country with this rare form of lung cancer visits this single specialist for their treatment. Being treated in this hospital for lung cancer is a collider, as it has two causes: living in the surrounding region, or having the rare carcinoid form. In reality, these two causes are independent: the risk of getting carcinoid lung cancer is the same for everyone, regardless of the region of residence. However, within the population of the patients treated in this hospital there appears to be a strong inverse relationship between living in this specific region and having carcinoid lung cancer. Patients who are treated in the hospital but are not from the surrounding region are very likely to have the rare form, whereas patients who live close to the hospital are very unlikely to have carcinoid lung cancer (namely 1%). This observed ‘spurious’ correlation is the result of conditioning on a collider through restricting the patient sample to only those treated in this single hospital. Including an indicator for being treated in this hospital as a regression variable in a multi-institutional study into lung cancer is another form of conditioning that will lead to similar collider bias.

We describe a fictional but realistic clinical scenario where the following conditions hold: (1) There exists a clinical need for outcome prediction. (2) This outcome partly depends on treatment, and an unbiased estimate of the treatment effect is required. (3) The DAG describing the data-generating process is assumed to be known. (4) An image is hypothesized to contain important information for the task in (1), however, one of the factors of variation in the image represents a collider in the DAG. Conditioning on this collider will lead to a biased estimate of (2). (5) The collider can be measured from the image. (6) Deep learning is used to optimally predict (1). We stress that this poses a conflicting problem: ‘simply’ using deep learning to predict the outcome based on the image may lead to a low prediction error of the outcome in the observed data, but it will lead to bias in the estimated effect of treatment, as it conditions on a collider. No matter how accurate the resulting predictions are on the observed data, such models cannot accurately predict in the setting where we intervene on treatment. This effectively nullifies the clinical usefulness of the model for selecting the best treatment for new patients. The model only ‘works’ when treatments are allocated as was always done without the model. On the other hand, ignoring the image all together will lead to worse prediction error as the image contains important prognostic information. Our contribution is that we show that by utilizing a multi-task prediction scheme for both the outcome and the collider, accompanied by an additional loss term to induce a form of linear independence between final layer activations, we can satisfy both (1) the supervised prediction task and (2) attain an unbiased estimate of the treatment effect. For clarity in notation, we will reserve the term prediction error for performance on the supervised prediction task (e.g., accuracy of predicted survival time). With bias we will refer to difference between the expectation of the estimated treatment effect and the data-generating mechanism.

## Results

### Clinical case

The proposed clinical case concerns the treatment of lung cancer. Optimal treatment selection for lung cancer patients is a challenging problem: depending on the clinical disease stage, patients receive (combinations of) chemotherapy, radiotherapy, surgery, or more recently, immunotherapy or targeted therapy.^2^ Some patients will be cured, while others only endure invalidating side-effects. In addition to using disease stage, personalized treatment decisions may be aided by estimating the individual prognosis of a patient for the different modes of treatment that are available. Medical scans provide important information for diagnosing and staging lung cancer, but may also provide this prognostic information. Deep learning is particularly attractive to analyze these scans, as these models may discover new prognostic factors or treatment effect modifiers.

### Data-generating mechanism

**Fig. 1:**
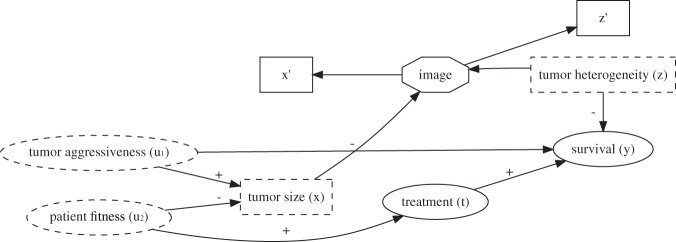
Directed Acyclic Graph describing the data-generating mechanism for the simulations. Signs indicate positive or negative associations. Rectangle shaped variables are image variables, dashed variables are unobserved. Tumor aggressiveness and patient fitness cannot be directly measured. $x,z$ represent biological processes, causing the outcome and image patterns. We cannot directly observe these biological processes, but $x^{\prime} ,z^{\prime}$ are noisy views of these variables that are measurable from the image. $x$ is a collider since it is the child of ${u}_{1}$ and ${u}_{2}$. Conditioning on $x$ will induce an artificial association between ${u}_{1}$ and ${u}_{2}$, thereby inducing a confounding path between treatment and survival, that only exists when conditioning on the collider.

In our experiments we use a public data set of chest CT-scans from the Lung Image Database Consortium image collection (LIDC^3^) These 1018 scans from 1010 unique patients each contain lung nodules ($N=2609$) suspected of lung cancer. Up to four radiologists segmented the nodules on each consecutive image slice. As described in the original publication of the data, the data where gathered from seven participating hospitals and the study was approved by the appropriate local institutional review boards (IRB). Informed consent procedures were followed according to local IRB guidelines, and the data collection and anonymization were conducted in compliance with the Health Insurance Portability and Accountability Act (HIPAA) guidelines with the intent of providing a publicly available data set. Our study is conducted in accordance with the usage guidelines from the data provider.^4^ We do not add new patient data, so IRB approval for this specific study was not needed. A CT-scan measures radiodensity, and tissues may exhibit different density-patterns. Heterogeneity in radiodensity is known to be associated with higher biologic aggressiveness and worse survival.^5^ We used nodule size and the variance of radiodensity in a simulation study involving a binary treatment and a real-valued outcome reflecting overall survival. Note that our simulation does not accurately reflect the real world. Real world applications would require more complex models. The aim of our contribution is to address a current limitation in methodological tools. Therefore we chose the simplest graphical model that induces the problem we try to solve, but is still clinically conceivable. A DAG used for a real-world clinical application will be much more complex, but may still include the basic collider structure we present in this simulation and will thus require similar methods. Figure 1 and Table 1 illustrate the following hypothetical narrative.

**Fig. 2:**
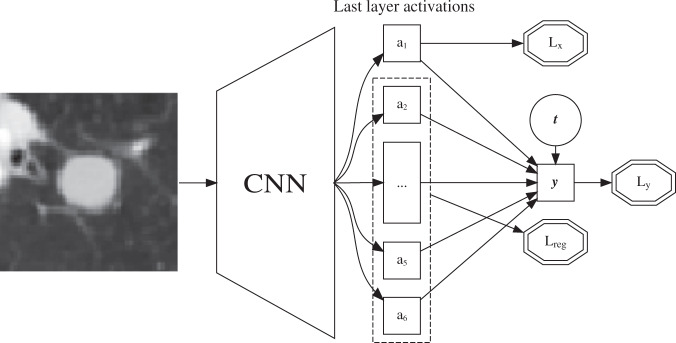
Schematic overview of the proposed convolutional neural network architecture. The network receives two inputs: an image and the treatment indicator ($t$). Loss functions are depicted in double octagons. The last layer activations are used to separate factors of variation in the image. ${a}_{1}$ is trained to approximate the measurement of the collider $x^\prime$. The rest of the last layer activations are constrained to be linearly independent from $x^\prime$ through ${L}_{{\mathrm{reg}}}$. The total loss is $L={L}_{y}+{L}_{x}+{L}_{{\mathrm{reg}}}$. CNN convolutional neural network.

**Table 1:** Parameters for sampling images and modeling outcome data.

	Variable	Variable model
${u}_{1}$	Aggressiveness	$N(0,0.7071)$
${u}_{2}$	Fitness	$N(0,0.7071)$
$z$	Heterogeneity	$N(0,1)$
$x$	Size	$N({u}_{1}-{u}_{2};0.05)$
$t$	Treatment	$Bern(invlogit(N({u}_{2}-0.5,0.25)))$
$y$	Survival	$N(t-z-2{u}_{1}-0.5;0.05)$

For each observation $i$, an image is drawn from the total pool of images with the closest ${x}_{i}$ and ${z}_{i}$. This ensures the required association between factors of variation in the image and the simulated outcome data. The parametric equations follow the DAG presented in Fig. 1: ${u}_{1},{u}_{2},z$ are continuous independent noise variables. The collider $x$ is the difference between ${u}_{1}$ and ${u}_{2}$, with a small amount of Gaussian noise (standard deviation of noise $=0.05$). ${u}_{1}$ and ${u}_{2}$ have a standard deviation of $0.7071\approx \sqrt{2}/2$ to ensure that $x$ has a standard deviation of $\approx 1$. Treatment $t$ is modeled as a Bernoulli variable with a logistic link function, where increased ${u}_{2}$ increases the probability of being treated. $0.5$ is subtracted to assure that ~$50 \%$ of patients are treated. Gaussian noise of standard deviation $0.25$ is added to the inverse log-odds of being treated to assure that every patient has some probability of being treated with the more intense treatment. This reflects the clinical world better as some patients may have strong preferences regarding their treatment, regardless of their underlying health status. Overall survival ($y$) increases with treatment (the true treatment effect is $1$) and decreases with heterogeneity in radiodensity and tumor aggressiveness. Again, Gaussian noise of standard deviation $0.05$ is added to introduce some uncertainty in the data

There exist two possible treatments for lung cancer: $t\in \{0,1\}$, where $t=1$ is deemed more aggressive and also more effective. An unobserved variable ${u}_{2}$ influences treatment allocation: people who appear to be in better overall health, as per subjective judgment of the physician, will have a higher probability of being treated with $t=1$. At the same time these fitter patients generally have a better functioning immune system. The immune system combats the lung cancer, leading to a lower tumor size ($x$). Another unobserved variable ${u}_{1}$ represents the tumor biologic aggressiveness. High aggressiveness leads to a bigger tumor and negatively impacts the overall survival. We emphasize that the tumor size ($x$) is a pre-treatment collider according to this causal graph. A third noise variable, heterogeneity of radiodensity ($z$), is a prognostic factor unrelated to the treatment, but related to the outcome. Tumors with high heterogeneity lead to reduced survival.

This situation leads to a conundrum. As can be seen from the DAG, the marginal average treatment effect is identified by ${\mathrm{ATE}}=E[p(y| t=1)-p(y| t=0)]$. The conditional treatment effect is not identified when conditioning the entire image, which is a descendant of both $x$ and $z$. Conditioning on $x^{\prime}$ (the tumor size as measured in the image), corresponds to partly conditioning the collider $x$. This will induce an artificial association between ${u}_{1}$ and ${u}_{2}$, thereby opening a confounding path from $t$ to $y$ and violating of the backdoor criterion.^1^ A backdoor path is a path from treatment to outcome that starts in the non-causal direction (an arrow pointing to the treatment instead of away from). This is indicative of confounding. When all confounding variables can be measured and conditioned on, all backdoor paths can be ‘closed’ during analysis, and the treatment effect can still be identified from observational data. In this case, a new backdoor path is introduced by conditioning on $x^{\prime}$, a proxy of $x$. This new path runs through the unobserved variables $t\leftarrow {u}_{1}-{u}_{2}\to y$. Therefore it cannot be closed by conditioning on these variables in the estimation, and the treatment effect is no longer identified. Using a convolutional neural network to predict $y$ without regard for the biasing effect of conditioning on the collider will lead to a biased estimate of the treatment effect. Disentangling the factors of variation in the image to only utilize image information that is not related to the collider would enable an unbiased estimate of the conditional treatment effect, which is the goal of this study. The simulated data are visualized in Supplementary Figs 1 and 2.

### Modeling

Our method, as summarized in Fig. 2, revolves around two central notions: (1) Utilizing the resemblance of the final layer of a CNN with linear regression and (2) Separating the contributions of different factors of variation during training to enable exclusion of factors of variation after model convergence. For each patient we have two observed quantities: ${y}_{i}\in {\mathbb{R}}$ and ${t}_{i}\in \{0,1\}$, along with an image which contains noisy views (${x}_{i}^{\prime},{z}_{i}^{\prime}\in {\mathbb{R}}$) of the tumor size ${x}_{i}$ and heterogeneity ${z}_{i}$. The tumor size ${x}_{i}$ is known to be a collider and can be measured from the image, tumor heterogeneity ${z}_{i}$ is an unknown prognostic factor that we expect a CNN can ‘discover’ by training it to predict survival. Following standard practice for predicting a continuous real outcome with deep learning, the last layer of the CNN resembles linear regression where $\hat{y}={\beta }_{0}+{\beta }_{t}t+{\sum }_{j=1}^{{N}_{k}}{\beta }_{j}^{k}{a}_{j}^{k}$, with ${a}_{j}^{k}$ the ${N}_{k}$ activations of the final layer of a $k$-layer CNN, $t$ the binary treatment indicator and ${\beta }_{0}$ an overall intercept. Indices for patients are omitted for clarity. Note that ${\beta }_{t}$ is the estimated average treatment effect (ATE). The standard minibatch mean squared error is used for $y$:

1 $${L}_{y}=\frac{1}{m}\sum _{i=1}^{m}{(\hat{y}-y)}^{2}$$

where $m$ the minibatch size. To attain separation of the collider from other factors of variation in the last layer, we modify the loss function such that a single activation of the last layer will approximate the collider: ${a}_{1}^{k}\approx x$. At the same time we optimize the other last layer activations $\{{a}_{j}^{k},j\,>\,1\}$ to be linearly independent of $x^\prime$. Note that this is a light constraint based on the prior knowledge represented in the DAG, namely that $x$ is a scalar and $x$ and $z$ are independent. We argue that after model convergence, we can fix all CNN parameters and do a single ordinary least squares on $\{{a}_{j}^{k}\cup t,j\,>\,1\}$ to get a valid estimate of the treatment effect with ${\beta }_{t}$. These activations are constrained to be linearly independent of the collider, so performing linear regression on these activations and the treatment indicator should mimic omitting the collider as a variable in the regression. To attain this, we add a loss term for the collider $x^\prime$:

2 $${L}_{x}=\frac{1}{m}\sum _{i=1}^{m}{({a}_{1}^{k}-x^\prime)}^{2}$$

This encourages the model to have a single activation in the last layer that approximates the collider $x$. This loss is synergistic with ${L}_{y}$ as predicting $x^\prime$ from the image will improve ${L}_{y}$ since $x$ and $y$ are statistically associated. At each training step, a prediction ${\hat{x}}^{{\mathrm{reg}}}$ is made by regressing $x^\prime$ on the remaining last layer activations $\{{a}_{j}^{k},\,j\,>\,1\}$ with ordinary least squares. The MSE of this regression measures how well $x^\prime$ can be predicted from a linear combination of the other last layer activations $\{{a}_{j}^{k},\,j\,>\,1\}$. This is compared with the MSE of predicting ${x^\prime}_{i}$ with $\bar{x}$, the mean of $x^\prime$ of that minibatch of patients. When predicting ${x^\prime}_{i}$ from $\{{a}_{j}^{k},\,j\,>\,1\}$ is no better than using the mean of $x^\prime$, these activations are sufficiently independent from $x$. When the converse is true, the difference in mean squared errors is added to the total loss.

3 $${L}_{{\mathrm{reg}}}:= {\mathrm{max}}(0,{\mathrm{MSE}}(\bar{x},x^\prime)-{\mathrm{MSE}}({\hat{x}}^{{\mathrm{reg}}},x^\prime))$$

The total loss is the direct sum of these losses.

4 $$L={L}_{y}+{L}_{x}+{L}_{{\mathrm{reg}}}$$

Training was continued until convergence or overfitting, as assessed by an increase in total loss on the independently simulated validation set with different images than in the training set. After convergence, all CNN parameters were fixed and the final layer activations were calculated for each image. A linear regression of $y$ was fitted on $\{{a}_{j},t;1\,<\, j\le {N}_{k}\}$ using the training set, resulting in a final model dubbed ‘CausalNet’.

### Experiments

We calculated three baseline models for comparison: (1) ignoring all image information and using only the treatment indicator, (2) linear regression on the ground truth data $\{t,x,z,y\}$ with (2) and without (3) conditioning on the collider $x$. Through the sampling scheme, along with ambiguity in manual nodule segmentations and limitations of statistical learning from finite data, there is inherent prediction error for $y$ and $x$. We estimated the MSE of this inherent error by predicting the ground truth labels $x$ and $z$ with a separate run of the same CNN architecture by replacing $y$ with $z$. For fair comparison of the methods, in the regression baseline models we replaced $x,z$ by $x^{\prime} ,z^{\prime}$ by adding gaussian noise to the simulated $x,z$ based on the MSE of the ground truth run for both variables. We compare the ‘curve fitting’ approach of conditioning on the entire image for predicting $y$ (BiasNet) with the proposed method (CausalNet). As presented in Table 2, the proposed method separates the biasing effect of the collider $x$ from the estimated treatment effect, and attains a prediction error close to the ideal expected loss for predicting $y$.

**Table 2:** Main results.

Model	Variables	${\mathrm{MSE}}_{{\mathrm{y}}}$	${\mathrm{ATE}}$
Regression	$t$	2.74	1.02
Regression	$t,x^{\prime} ,z^{\prime}$	1.39	0.65
Regression*	$t,z^{\prime}$	1.99	1.00
BiasedNet	$t,image$	1.83	0.66
CausalNet	$t,{a}_{j}^{k}(j\,>\,1)$	2.23	1.02

Mean squared error for survival (MSE_y_) along with estimated average treatment effect (ATE). The linear regression metrics are the expected outcomes according to whether or not the model conditions on the collider $x$. Regression* is the optimal value for our setup: (1) predicting the outcome based on relevant prognostic information from the image while (2) retaining a valid estimate of the treatment effect. All metrics were calculated on the validation set

### Measurement error

To test the sensitivity of our method to measurement error in the measured collider $x$, we simulated two additional scenarios where the collider was measured on the wrong scale. In one scenario, the actual relationship between the collider and the outcome was linear in the diameter of the nodule, while it was measured in units of volume. This represents a power 3 mismatch between the measurement and the actual relationship. The inverse scenario was studied as well. See Supplementary Fig. 3 for a visualization of relationship the measured $x^\prime$ and the true $x$. As shown in Table 3, the method seems robust to these kinds of measurement errors.

**Table 3:** Sensitivity analysis to measuring the collider on the wrong scale.

Model	Actual $x$	Measured $x^\prime$	${\mathrm{MSE}}_{{\mathrm{y}}}$	${\mathrm{ATE}}$
Regression*	Area	Area	1.99	1.00
CausalNet	Area	Area	2.23	1.02
CausalNet	Diameter	Volume	2.24	0.99
CausalNet	Volume	Diameter	2.21	1.02

Mean squared error for survival (MSE_y_) along with estimated average treatment effect (ATE). The regression* results indicate the optimal results attainable for this simulated scenario

## Discussion

We provide a realistic medical example where plain curve fitting with deep learning will lead to biased predictions that do not generalize to the setting where we intervene on treatment. By utilizing prior knowledge about the world in the design of the CNN architecture and optimization scheme, accurate survival predictions were feasible with an unbiased estimate of the treatment effect. Our experiments demonstrate that deep learning can in principle be combined with insights from causal inference. Possible directions for extension of our experiments are introducing more elaborate data-generating mechanisms, for example with a treatment effect modifier or with statistical dependence between factors of variation within the image. In addition, similar approaches can be explored for medical images from different sources (e.g., pathology slides), or different data domains such as audio or natural language. We leave these extensions for further work.

Real world clinical applications of causal inference will necessarily involve more complicated DAGs. These DAGs could include one or more colliders. Our method can be adapted to multiple colliders in a straightforward manner by reserving a last layer activation for each collider, and requiring the other last layer activations to be independent of each of these colliders. Each real-world clinical scenario will require its own DAG for identifying treatment effects from observational data. Our contribution is that the proposed method can be used to attain deep representations of images that are independent of certain factors of variation.

Aside from the mitigation of collider bias, the proposed method can possibly be useful for other applications. For example, it may be used to produce deep representations of CT-scans that are independent of the scanner vendor. The scanner vendor would then take the place of the collider $x$ in our simulation example.

To attain the goal of personalized treatment recommendations with artificial intelligence, methods combining machine learning with causal inference need to be further developed. Our experiments provide an example of how deep learning and structural causal models can be combined and are a small step forward towards personalized health care.

## Methods

### Data preparation and simulation

The LIDC-IDRI data set provides 1018 scans from 1010 patients with a total of 2609 nodules. The nodules were split in a training (70%) and validation (30%) set. Individual slices of the nodules were extracted and size (pixel count within segmentation) and heterogeneity (variance of pixel intensities within the segmented nodule) were calculated for each of the slices. Slices with a nodule size of $<20\,{\mathrm{mm}}^{2}$ were removed, as well as slices for which not all annotators agreed on the presence of a nodule. This yielded a training pool of 5015 slices and a validation pool of 1528 slices. Observations were simulated by sampling noise variables from the appropriate distributions and dependent variables according to the structural causal model in Table 1. For each patient $i$ with simulated ${x}_{i},{z}_{i},{t}_{i},{y}_{i}$, an image was drawn with replacement from the corresponding pool of images with the closest measured $x^{\prime}$ (size) and $z^{\prime}$ (heterogeneity). This sampling procedure induces a controllable statistical association between patterns in the image and the simulated treatment and outcome data. We simulated 3000 training observations and 1000 validation observations. Square slices of 7 $\times$ 7 cm surrounding the nodules were extracted from the CT-slices and resampled to isotropic 0.7 mm spacing. Pixel intensities were normalized to unit scale using a global mean and variance. The images were cropped randomly to 51 $\times$ 51 pixels during training, center crops of the same size were used for validation. In addition, random vertical and horizontal mirroring was used as data augmentation during training.

### Neural network

We employed a VGG-like^6^ CNN architecture. As our aim is to contrast methods of optimization for attaining unbiased predictions, we chose a simple CNN architecture with only basic layer types that was small enough for fast training but expressive enough to be able to model the nodule size and heterogeneity. The final network consisted 5 layers of 3 $\times$ 3 convolutions with 16 feature channels, each followed by a ReLU non-linearity and 2 $\times$ 2 max-pooling. These basic image features were flattened into a 1 dimensional vector of size 144. Three fully connected layers of output sizes 144, 144, 12 were used, each followed by ReLU and dropout with $p=0.25$, after which a final fully connected layer with output size ${N}_{k}=6$ was used. The treatment indicator was concatenated to these activations for the final prediction during training. We used a batch size of 40 and the Adam optimizer^7^ with a learning rate of 0.001 and no weight-decay.

### Reporting summary

Further information on research design is available in the Nature Research Reporting Summary linked to this article.

## Supplementary information

reporting summary

supplemental material

## Data Availability

The image data used in this study are publicly available through the Cancer Imaging Archive repository (10.7937/K9/TCIA.2015.LO9QL9SX). The outcome data were generated randomly according to the data-generating mechanism described in Table 1.

## Code availability

The source code for replicating the experiments is made available freely online under the MIT open source license. The code includes the pre-processing of the original CT-scans, simulation of observations and running of experiments. Source code link: 10.5281/zenodo.3522229
