# Supplementary material for: Eliminating biasing signals in lung cancer images for prognosis predictions with deep learning
Source: NPJ Digit Med. 2019 Dec 10;2:122. doi: 10.1038/s41746-019-0194-x (PMC6904461; doi:10.1038/s41746-019-0194-x)
Supplement: Supplementary file 2 — supplemental material [file 41746_2019_194_MOESM2_ESM.pdf]

Supplemental Data: Eliminating Biasing Signals in Lung Cancer Images for Prognosis Predictions with Deep Learning

W.A.C. van Amsterdam*[1], J.J.C. Verhoeff[2], P.A. de Jong[1], T. Leiner[1], and M.J.C. Eijkemans[3]

[1] Department of Radiology,
[2] Department of Radiotherapy,
[3] Biostatistics and Research Support, Julius Center for Health Sciences and Primary Care,
University Medical Center Utrecht, Utrecht, The Netherlands,
*w.a.c.vanamsterdam@umcutrecht.nl

October 30, 2019

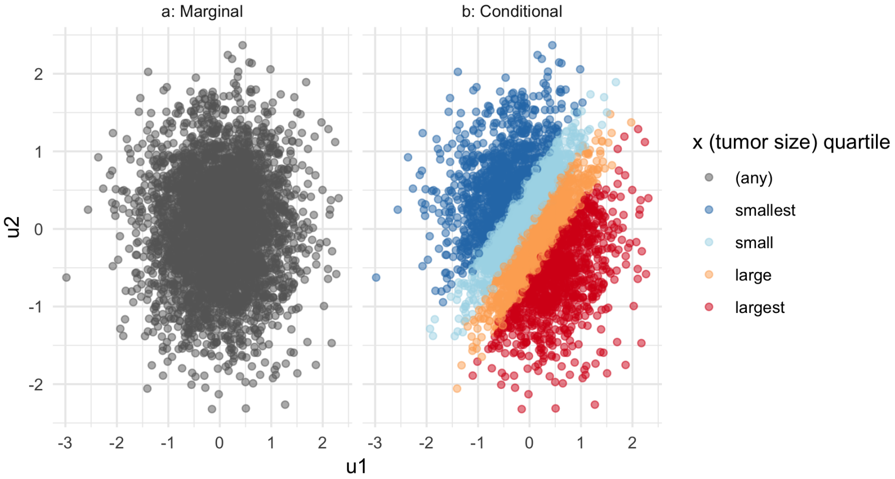

Supplementary Figure 1: Visualization of the artificial correlation induced by conditioning on the collider x. Through the data generating mechanism, u_1 and u_2 are independently generated Gaussian variables. Facet a shows that the variables are marginally independent. The collider x is simulated as the difference between u_1 and u_2 with some Gaussian noise ($x \sim N(u_1 - u_2, 0.05)$). This means that for any given value of x, u_1 and u_2 are positively correlated. Facet b visualizes this artificial correlation by binning the values of x for the simulated data in quartiles.

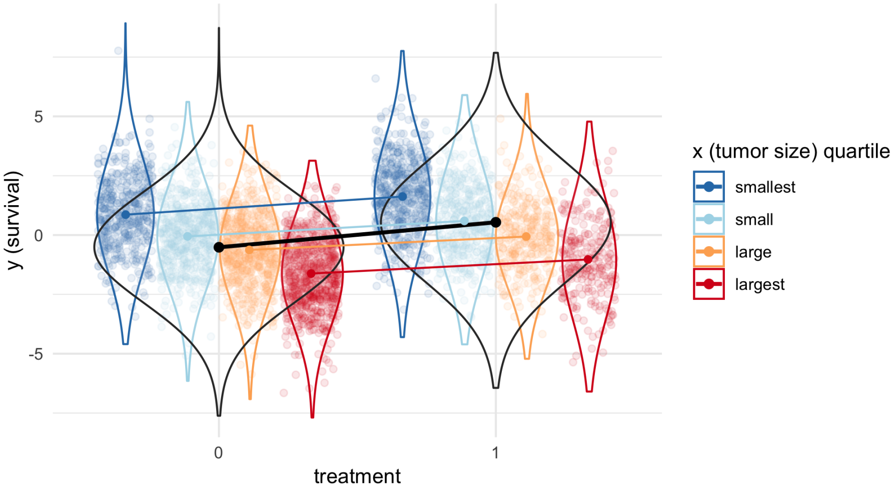

Supplementary Figure 2: Visualization of the biasing effect of conditioning on the collider x (tumor size). The true treatment effect is 1. The solid black line visualizes the true (causal) difference in y (survival) between treated an untreated patients when **not** conditioning on the collider x. When observations are conditioned on the collider x, visualized here by binning patients in quartiles of x, the observed difference in survival between treated and untreated patients diminishes, as indicated by the colored lines. This diminished difference in survival between patients occurs when conditioning on the collider x. Conditioning on x induces a positive correlation between its parents u_1 and u_2. u_1 increases the probability of intensive treatment, while u_2 decreases the probability of survival. Due to this artificial association between u_1 and u_2, induced by conditioning on the collider x, the difference in survival between treated and untreated patients appears diminished. In reality, **assigning** a patient to intensive treatment will always increase their survival with 1, as reflected in the black line.

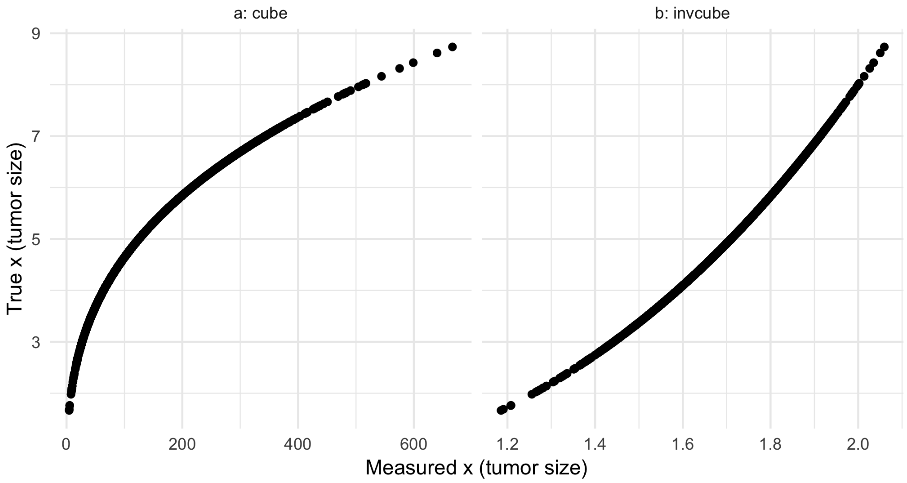

Supplementary Figure 3: Visualization of collider measurement and actual value. Facet a: x is measured as diameter, while y is linear in volume of the tumor Facet b: x is measured by volume, while y is linear in the diameter of the tumor.
